# Usefulness of interferon-γ release assay for the diagnosis of sputum smear-negative pulmonary and extra-pulmonary TB in Zhejiang Province, China

**DOI:** 10.1186/s40249-017-0331-1

**Published:** 2017-09-01

**Authors:** Lei Ji, Yong-Liang Lou, Zhong-Xiu Wu, Jin-Qin Jiang, Xing-Li Fan, Li-Fang Wang, Xiao-Xiang Liu, Peng Du, Jie Yan, Ai-Hua Sun

**Affiliations:** 1 Department of Basic Medicine, Hangzhou Medical College, Hangzhou, 310053 Zhejiang People’s Republic of China; 2 School of Laboratory Medical Science and Life Science, Wenzhou Medical University, Wenzhou, 325035 Zhejiang People’s Republic of China; 3 Zhejiang Blood Center, Hangzhou, 310006 Zhejiang People’s Republic of China; 4 Division of Basic Medical Microbiology, State Key Laboratory for Diagnosis and Treatment of Infectious Diseases, First Affiliated Hospital of Zhejiang University, Hangzhou, 310003 Zhejiang People’s Republic of China; 5 Department of Medical Microbiology and Parasitology, College of Medicine, Zhejiang University, Hangzhou, 310058 Zhejiang People’s Republic of China

**Keywords:** Interferon-gamma release assay, Smear-negative pulmonary TB, Extra-pulmonary TB

## Abstract

**Background:**

Quick diagnosis of smear-negative pulmonary tuberculosis (TB) and extra-pulmonary TB are urgently needed in clinical diagnosis. Our research aims to investigate the usefulness of the interferon-γ release assay (IGRA) for the diagnosis of smear-negative pulmonary and extra-pulmonary TB.

**Methods:**

We performed TB antibody and TB-IGRA tests on 389 pulmonary TB patients (including 120 smear-positive pulmonary TB patients and 269 smear-negative pulmonary TB patients), 113 extra-pulmonary TB patients, 81 patients with other pulmonary diseases and 100 healthy controls. Blood samples for the TB-Ab test and the TB-IGRA were collected, processed, and interpreted according to the manufacturer’s protocol.

**Results:**

The detection ratio of smear-positive pulmonary TB patients and smear-negative pulmonary TB patients were 90.8% (109 of 120) and 89.6% (241 of 269), respectively. There was no statistically significant difference of its performance between these two sample sets (*P* > 0.05). The detection ratio of positive TB patients and extra-pulmonary TB patients were 90.0% (350 of 389) and 87.6% (99 of 113), respectively, which was not significantly different (*P* > 0.05).

**Conclusions:**

In this work, the total detection ratio using TB-IGRA was 89.4%, therefore TB-IGRA has diagnostic values in smear-negative pulmonary TB and extra-pulmonary TB diagnosis.

**Electronic supplementary material:**

The online version of this article (doi:10.1186/s40249-017-0331-1) contains supplementary material, which is available to authorized users.

## Multilingual abstracts

Please see Additional file 1 for translations of the abstract into five official languages of the United Nations.

## Background

The World Health Organization’s (WHO) flagship Global TB Report 2016, released on Oct 12, considers *Mycobacterium tuberculosis* as one of the most significant human pathogens that are harmful to the global public health [1]. Over 550 million Chinese are infected with *M. tuberculosis*, including nearly 100 million extra-pulmonary tuberculosis (TB) patients and 300 million sputum smear-negative and/or culture-negative infections [1]. The clinical manifestations of TB are highly varied and unspecific; *M. tuberculosis* is able to infect various systems of the human body. The bacteriological examination method (smear microscopy) is still the gold standard for TB diagnosis in many countries, but the low smear-positive rate and long incubation period required to obtain positive cultures are problematic for the requirements in clinical diagnosis and treatment. Extra-pulmonary disease, like disseminated TB, brings great difficulties in clinical diagnosis [2, 3], since it is very hard to detect *M. tuberculosis* in patient’s sputum. Therefore, it results in a delay in the diagnostic process for pulmonary TB, especially in smear-negative cases [4]. Early detection and accurate diagnosis of TB could reduce the morbidity and spread of TB and it is also the key element in TB control [4]. Serological diagnosis of TB, such as the TB antibody (TB-Ab) test, is widely used in the laboratory in China, but the diagnostic yield is generally no more than 70%. Therefore, there is an urgent need for developing rapid and highly sensitive technology to change this situation [5, 6]. The interferon-γ release assay (IGRA) uses a special antigen as stimulus and follows Enzyme Linked Immunosorbent Assay (ELISA) procedures for detection has potentially high specificity and sensitivity. After being invented by Lalvani [5], this method is widely used in many counties and has become the most commonly used diagnostic method in TB detection [7–10].

IGRA is an ex vivo blood test of the T-cell immune response. It detects the T-cell release of interferon-gamma (IFN-γ), an inflammatory cytokine, following stimulation by antigens specific to the *M. tuberculosis* complex. These antigens include 6-kDa early secretory antigenic target (ESAT-6) and 10-kDa culture filtrate antigen (CFP-10), which are more specific for *M. tuberculosis* than the tuberculin skin test (TST) because they are not produced by Bacillus Calmette–Guérin (BCG) vaccine strains and non-tuberculosis mycobacteria (NTM) strains [11].

In order to verify the sensitivity and specificity of TB-IGRA in TB tests, we compared the diagnostic performance of TB-IGRA and TB-Ab tests in 389 pulmonary TB patients, 113 extra-pulmonary TB patients, 81 patients with other pulmonary diseases and 100 healthy individuals visiting the Center of Physical Examination for routine physical examination.

## Methods

### Subjects

This work has obtained approval from the Research Ethics Committee of Hangzhou Medical College. All subjects signed the informed consent forms as approved. Demographic and clinical data were collected using a specific form and then transferred into a database. The examination for TB was usually completed within one week. Whole blood samples of 502 patients with TB were collected from Hangzhou Red Cross Hospital, Quzhou People’s Hospital, and Hospital of integrated traditional Chinese and Western medicine in Xiacheng District of Hangzhou and the Center for Disease Control and Prevention (CDC) of Zhejiang Province of China between September 2012 and June 2013. The demographic characteristics of the subjects in this study are shown in Table 1. The average age of the patients was 45 years and ranged from 13 to 82 years of age.

**Table 1:** The demographic characteristics of the subjects

Population	No.	M/F ^a^	Median (IQR) age (years)
Smear-positive pulmonary TB patients	120	77/43	45(25–61)
Smear-negative pulmonary TB patients	269	173/96	
Extra-pulmonary TB patients	113	66/47	
Other lung diseases patients	81	28/53	49(33–61)
Healthy controls	100	56/44	48(44–53)

^a^ M/F indicates the ratio of male/female

According to sputum-smear, sputum-culture and X-ray tests, 120 patients were classified as smear-positive pulmonary TB patients, 269 patients were classified as smear-negative pulmonary TB patients and 113 patients were classified as extra-pulmonary TB patients (including tuberculous pleurisy, tuberculous peritonitis, spinal TB, renal TB, pelvic TB and intestinal TB). According to clinical manifestations, pathological diagnosis, sputum smear and culture, and X-ray diagnosis, 81 patients were classified as other lung disease patients (including pneumonia, lung cancer, lung tumors and bronchiectasis); the average age of these patients was 49 years and ranged from 17 to 82 years of age. One hundred cases of healthy individuals visiting the Center of Physical Examination for routine physical examination were collected from the above-mentioned hospitals. These healthy individuals had no prior history of TB and were sputum culture and X-ray negative. The average age of these subjects was 48 years and ranged from 31 to 60 years of age.

### Reagents and instruments

TB-IGRA kits and TB-Ab kits were purchased from Beijing Wantai Biological Pharmacy Co., Ltd. and Nanjing Potomac Bio-Technology Co., Ltd., respectively. Multiskan MK-3 from Lab systems. Biosafety Cabinet from Heal Force. BD-TEK-ELX50 from Bio-Tek Company.

### TB-Ab test

The TB-Ab test was used to detect serum samples. Assays and interpretation of results were strictly according to the manufacturers’ instructions. Simultaneously, sputum smear and sputum culture assays were performed.

### Interferon-γ release assay

All subjects were tested with TB-IGRA according to the manufacturer’s instructions. Six milliliter fresh whole blood was collected using heparin vacutainer blood collection tubes. Briefly, 1 ml of peripheral venous blood samples collected from each participant were injected in three special culture tubes for the TB-IGRA: One test tube (T) coated with *M. tuberculosis*-specific antigens (a recombinant fusion protein of CFP-10 and ESAT-6), one positive control tube (P) containing phytohemagglutinin (PHA), and one negative control tube (N). Then the three tubes were incubated at 37 °C for 22 ± 2 h. Subsequently, the tubes were centrifuged at 3000 g for 10 min and the serum was stored at −20 °C until detection. The IFN-γ levels were detected using the ELISA method. Positive samples were identified following the criteria defined in Table 2 [12].

**Table 2:** TB-IGRA detection criterion

N	P – N	T – N	Results
≤ 400	Any value	≥ 14 and ≥ $\frac{N}{4}$	Positive
	≥ 20	< 14	Negative
		≥ 14 but < $\frac{N}{4}$	
	< 20	< 14	Uncertain
		≥ 14 but < $\frac{N}{4}$	
≥ 400	Any value	Any value	

*T* Testing tube (T) content value, *N* Negative control tube (N) content value, *P (Unit: pg/ml)* Positive control tube (P) content value

### Statistical analysis

Statistical analysis was performed using IBM SPSS Statistics ver. 11.0 (IBM Co., Armonk, NY, USA). Statistical significance was defined as *P* < 0.05. Student’s *t*-tests were used to compare continuous variables of the study groups. To evaluate discrete variables, Pearson chi-square and Fisher exact tests were performed as appropriate. For the assessment of diagnostic agreement between tests, Cohen κ statistics were used.

## Results

### TB-IGRA test results of different patient groups

The total detection ratio of TB-IGRA tests for all groups combined was 89.4% (449 of 502), while the detection ratios of the TB group and extra-pulmonary TB group were 90.0% (350 of 389) and 87.6% (99 of 113), respectively. The detection ratios of sputum positive and smear-negative TB patient groups were 90.8% (109 of 120) and 89.6% (241 of 269), respectively, and the detection ratios between these two patient groups were not significant different (*χ* ^2^ = 0.52 and 0.14,*P* > 0.05). The detection ratios of TB-IGRA in other lung diseases and healthy controls was 11.6% (21 of 181), while the non-TB lung disease group TB-IGRA total detection ratio was 16.0% (13 of 81). Therefore, it was significantly lower than the detection ratio in TB patients, which was 90.0% (350 of 389) (*χ* ^2^ = 208.36, *P* < 0.01) (Tables 3 and 4).

**Table 3:** Sensitivity comparison of different testing methods for TB patients

Methods	TB, *n* = 389						Extra-pulmonary TB, *n* = 113			
	Culture Positive, *n* = 120		Culture negative, *n* = 269		Total, *n* = 389					
	P	S (%)	P	S (%)	P	S (%)	P	S (%)	P	S (%)
TB-IGRA	109	90.8	241	89.6	350	90.0	99	87.6	449	89.4
TB-Ab	50	41.7	85	31.6	135	34.7	35	31.0	170	33.9

*P* short for Positive cases, *S* short for Sensitivity

**Table 4:** Specificity comparison of different methods

Methods	Other pulmonary diseases, *n* = 81		Healthy controls, *n* = 100		Total, *n* = 181	
	P	S (%)	P	S (%)	P	S (%)
TB-IGRA	13	83.9	8	92.0	21	88.4
TB-Ab	11	86.4	7	93.0	18	90.1

*P* short for Positive cases, *S* short for Sensitivity

### Sensitivity comparison of TB-IGRA and TB-Ab test

The TB-IGRA and TB-Ab detection sensitivities of sputum positive TB patients were 90.8% (109 of 120) and 41.7% (50 of 120), respectively, the detection sensitivities of smear-negative TB patients were 89.6% (241 of 269) and 31.6% (85 of 269), respectively, and the overall detection sensitivities of TB patients were 90.0% (350 of 389) and 34.7% (135 of 389), respectively. The TB-IGRA and TB-Ab detection sensitivities of extra-pulmonary TB were 87.6% (99 of 113) and 31.0% (35 of 113), respectively, while the overall sensitivities of TB-IGRA and TB-Ab detection of TB were 89.4% (449 of 502) and 33.9% (170 of 502), respectively. Therefore, the sensitivity of the TB-IGRA test was significantly higher than the sensitivity of the TB-Ab test (*χ* ^2^ = 64.9–327.9, *P* < 0.01) (Table 3).

### Specificity comparison of TB-IGRA and TB-Ab test

The specificity of the TB-IGRA tests for the healthy control group and the other lung diseases group were 92.0% (92 of 100) and 83.9% (68 of 81), respectively, while the specificity of the TB-Ab tests for the healthy controls group and the lung diseases group were 93.0% (93 of 100) and 86.4% (70 of 81), respectively. The overall specificity of the TB-IGRA tests and the TB-Ab tests were 88.4% (160 of 181) and 90.1% (163 of 181), respectively, which were not significantly different (*χ* ^2^ = 0.2, *P* > 0.05) (Table 4).

## Discussion

Currently, rapid diagnosis of TB is depending on observation of clinical symptoms, radiographic examination, smears and culture of clinical samples [4]. Because the negative rate of smears and culture of clinical samples is too high and the pathological samples for testing are hard to obtain, it is difficult to diagnose smear-negative and extra-pulmonary TB. Although the TST test is widely used to detect TB, NTM and BCG vaccination is known to introduce false-positive results [5]. Furthermore, in the TST test, patients with advanced HIV disease could give rise to false negative results [6]. In China, the BCG vaccination program has been widely implemented and causes a highly false-positive detection ratio in TST test [13]. Therefore, immunological methods with high sensitivity and specificity to detect smear-negative and extra-pulmonary TB are urgently needed.

In this work, the total detection ratio of TB-IGRA tests was 89.4%, which was higher than the widely used TB-Ab test that showed a detection ratio of 33.9% (*P* < 0.01). Furthermore, these ratios were also higher than results reported by Wang JY (87%) [14] and Kobashi Y (86%) [15], but slightly lower than Kim SH’s result (95%) [16]. Overall, TB-IGRA showed a higher detection ratio of TB than other serological methods. In addition, the detection ratio of this method in smear-negative TB patients and positive pulmonary TB patients showed no significant difference (89.6% vs 90.8%, *P* > 0.05). Given that the TB-IGRA test has a high sensitivity, is able to detect smear-negative patients effectively, and can be used for latent TB detection, we used TB-IGRA for the diagnosis of TB and extra-pulmonary TB. The sensitivity of these tests were 90.0 and 87.6%, which was not significantly different (*P* > 0.05). The sensitivity of the TB-Ab test that is used in TB and extra-pulmonary TB detection was 34.7 and 31%, which was significantly lower than the sensitivity of TB-IGRA (*P <* 0.01). Furthermore, the TB-IGRA and TB-Ab specific detection ratios of healthy controls were 92.0 and 93.0%, while specific detection ratios in the lung disease group were 83.9 and 86.4%. Therefore, no significant difference in specificity was observed (*P* > 0.05). Although the detection ratio of the TB-IGRA was very high, still 8 positive samples in the healthy controls group were detected. Therefore, we need to confirm whether these positive samples are real false positives or latent TB.

## Conclusions

In this work, we showed that the total detection ratio of the TB-IGRA tests was 89.4%, confirming that the TB-IGRA test has high sensitivity and specificity and diagnostic values in smear-negative pulmonary TB and extra-pulmonary TB diagnosis. The use of the TB-IGRA test may help in the early detection and accurate diagnosis of TB.

## Additional file

Additional file 1: Multilingual Abstracts in the five official working languages of the United Nations. (PDF 869 kb)

## Abbreviations

BCG: Bacillus Calmette–Guérin
CDC: Centers for disease control and prevention
CFP-10: 10 kDa culture filtrate antigen
ELISA: Enzyme linked immunosorbent assay
ESAT-6: 6-kDa early secretory antigenic target
IFN-γ: Interferon-γ
IGRA: Interferon-γ release assay
NTM: Non-tuberculosis mycobacteria
PHA: Phytohemagglutinin
TB: Tuberculosis
TB-Ab: TB antibody
TST: Tuberculin skin test
WHO: World Health Organization
