# Supplementary material for: Usefulness of interferon-γ release assay for the diagnosis of sputum smear-negative pulmonary and extra-pulmonary TB in Zhejiang Province, China
Source: Infect Dis Poverty. 2017 Sep 1;6:121. doi: 10.1186/s40249-017-0331-1 (PMC5579952; doi:10.1186/s40249-017-0331-1)
Supplement: Additional file 1: — Multilingual Abstracts in the five official working languages of the United Nations. (PDF 869 kb) [file 40249_2017_331_MOESM1_ESM.pdf]

Translation of the abstract into the five official working languages of the United Nations

الفائدة من فحص افراز الجاما الإنترفيرون لتشخيص مسحة البلغم الرئوى و خارج الرئوي السلبية للسل في مقاطعة تشجيانغ، الصين

Lei Ji, Yong-Liang Lou, Zhong-Xiu Wu, Jin-Qin Jiang, Xing-Li Fan, Li-Fang Wang, Xiao-Xiang Liu, Peng Du, Jie Yan and Ai-Hua Sun

المستخلص

المقدمة: هناك حاجة ملحة و سريعة لتشخيص السل الرئوي والسل خارج الرئة السلبي بواسطة تحليل مسحة البلغم في التشخيص السريري. ويهدف بحثنا إلى التحقيق في فائدة فحص الجاما إنتيرفيرون لتشخيص السل الرئوي والسل خارج الرئوي الذى يعطى نتيجة سالبية بواسطة تحليل مسحة البلغم

الطريقة: أجرينا تحاليل الاجسام المضادة للسل (ت ب- أب) و ت ب-إيغرا على 389 مريضا بالسل الرئوي (بما في ذلك 120 مريضا مصابا بالسل الرئوي إيجابي المسحة و 269 مريضا بالسل الرئوي السلبي المسحة)، و 113 مريضا بالسل خارج الرئوي، و 81 مريضا يعانون من أمراض رئوية أخرى، و 100 شخص سليم صحيا كضوابط. تم جمع عينات الدم من اختبار ت ب-أب و ت ب-إيغرا ومعالجتها وتفسيرها وفقا لبروتوكول الشركة الصانعة

النتائج: كانت نسبة اكتشاف مرضى السل الرئوي الإيجابي المسحة ومرض السل الرئوي السلبي المسحة 90.8٪ (109 من 120) و 89.6٪ (241 من 269) على التوالي. لم يكن هناك فرق ذو دلالة إحصائية في أدائها بين مجموعتي العينة (P> 0.05). وكانت نسبة الكشف عن مرضى السل الإيجابيين ومرضى السل خارج الرئتين 90.0٪ (350 من 389) و 87.6٪ (99 من 113)، على التوالي، والتي لم تختلف معنويا (P> 0.05)

الملخص: في هذا العمل، كانت نسبة الكشف الكلي باستخدام تب-إيغرا 89.4٪، وبالتالي تب-إيغرا لديها قيم تشخيصية في السل الرئوي السلبي المسحة والتشخيص السل خارج الرئوي.

Translated from English version into Arabic by Khaled Zayed

干扰素体外释放酶联免疫法在痰涂片阴性肺结核和肺外结核诊断中的应用价值

Lei Ji, Yong-Liang Lou, Zhong-Xiu Wu, Jin-Qin Jiang, Xing-Li Fan, Li-Fang Wang, Xiao-Xiang Liu, Peng Du, Jie Yan and Ai-Hua Sun

摘要

引言：痰涂片阴性肺结核和肺外结核的快速诊断是临床诊断的一个重要问题，本研究旨在探讨干扰素体外释放酶联免疫法(IFN-γ release assay, IGRA) 在痰涂片阴性肺结核和肺外结核诊断中的应用价值。

方法：用 TB-Ab 试剂盒和 TB-IGRA 试剂盒分别检测 389 例肺结核病患者（包括 120 例痰涂片阳性肺结核患者和 269 例痰涂片阴性肺结核患者）和 113 例肺外结核，81 例其他肺部疾病患者和 100 例健康对照者结核感染情况。血液样本的采集、处理和结果判断均严格按照两种试剂盒的说明操作。

结果：TB-IGRA 检测痰涂片阳性和痰涂片阴性肺结核患者的阳性检出率分别为 90.8% (109/120) 和 89.6% (241/269)，两者之间无显著性差异（$P > 0.05$）；TB-IGRA 检测肺结核和肺外结核的阳性检出率分别为 90.0% (350/389)和 87.6% (99/113)，两者之间无显著性差异（$P > 0.05$）。

结论：TB-IGRA 在本研究中的阳性检出率为 89.4%，用该方法检测痰涂片阴性肺结核和肺外结核具有快速和较高的灵敏度和特异性，值得临床推广应用。

Translated from English version into Chinese by Lei Ji

Utilité du test de détection de production d'interféron-γ pour le diagnostic de la tuberculose pulmonaire à frottis négatif de crachat et de la tuberculose extra-pulmonaire dans la Province de Zhejiang, Chine

Lei Ji, Yong-Liang Lou, Zhong-Xiu Wu, Jin-Qin Jiang, Xing-Li Fan, Li-Fang Wang, Xiao-Xiang Liu, Peng Du, Jie Yan and Ai-Hua Sun

Résumé

Contexte: La détection rapide de la tuberculose pulmonaire à frottis négatif et de la tuberculose extra-pulmonaire est de toute urgence pour le diagnostic clinique. Notre recherche visait à étudier l'utilité du test sanguin de détection de production d'interféron-γ (IFN-γ) pour le diagnostic de la tuberculose pulmonaire à frottis négatif et extra-pulmonaire.

Méthode: Nous avons effectué des tests de détection d'anticorps et de production d'IFN-γ pour tuberculose sur 389 patients atteints de tuberculose pulmonaire (dont 120 patients à frottis positif et 269 patients à frottis négatif), 113 patients à tuberculose extra-pulmonaire, 81 patients atteints d'autres maladies pulmonaires et 100 individus sains. Des échantillons de sang pour les tests de detection d'anticorps et de production d'IFN-γ pour tuberculose ont été collectés, traités et interprétés selon le protocole du fabricant.

Résultat : Le taux de détection de la tuberculose pulmonaire chez les patients à frottis positif et à frottis négatif était de 90,8% (109 sur 120) et 89,6% (241 sur 269), respectivement. Il n'y a pas de différence statistiquement significative de performance entre ces deux ensembles d'échantillons ($P > 0,05$). Le taux de détection chez les patients à tuberculose pulmonaire et extra-pulmonaire était de 90.0% (350 sur 389) et 87.6% (99 sur 113), respectivement, ce qui n'était pas significativement différente ($P > 0,05$).

Conclusion: Dans cette étude, le taux total de dépistage utilisant le test de détection de prodution d'IFN-γ était de 89,4% et, par conséquent, le test de détection de prodution d'IFN-γ est d'une grande valeur pour le diagnostic de la tuberculose pulmonaire à frottis négatif et de la tuberculose extra-pulmonaire.

Translated from English version into French by Kokouvi Kassegne

Полезность интерферона-γ выпускает тест для диагностики микроскопии мокроты легочного и внелегочного туберкулеза в провинции Чжэцзян, Китай

Lei Ji, Yong-Liang Lou, Zhong-Xiu Wu, Jin-Qin Jiang, Xing-Li Fan, Li-Fang Wang, Xiao-Xiang Liu, Peng Du, Jie Yan and Ai-Hua Sun

Аннотация

Введение: Быстрая диагностика с отрицательным мазком туберкулеза легких и внелегочного туберкулеза срочно необходимы в клинической диагностике. Наше исследование состоит в том, чтобы исследовать полезность выпуска теста интерферона-γ для диагностики мазка-отрицательного легочного и внелегочного туберкулеза.

Методы исследования: Мы провели антитела к TB и TB-IGRA тесты для 389 больных с туберкулезом легких (в том числе 120 бациллярных больных с туберкулезом легких и 269 больных с мазком отрицательным мокроты туберкулезом легких), 113 больных с внелегочноым туберкулезом, 81 больных с другими легочными заболеваниями и 100 здоровых. Анализы крови для теста туберкулез-Ab и TB-IGRA были собраны, обработаны и интерпретированы согласно протоколу производителя.

Результаты исследования: Коэффициент обнаруживания бациллярных больных туберкулезом легких и больных с мазком отрицательным мокроты туберкулезом легких были 90.8% (109 из 120) и 89,6% (241 из 269) соответственно. Не было статистически значимой разницы производительности между этими двумя наборами образцов ($p > 0,05$). Коэффициент обнаружения положительных больных туберкулезом и больных туберкулезом легких составллял 90,0% (350 из 389) и 87,6% (99 из 113) соответственно, что достоверно не отличалось ($p > 0,05$).

Заключение: В данной работе коэффициент обнаружения с помощью TB-IGRA был 89.4%, поэтому TB-IGRA имеет диагностическое значение в диагностике мазка-негативного туберкулеза легких и внелегочного туберкулеза.

Translated from English version into French by Hao-Qi Zhang

Utilidad del ensayo de liberación de interferón-γ para el diagnóstico de TB de frotis negativa pulmonar y extrapulmonar basado en esputo en la provincia de Zhejiang, China

Lei Ji, Yong-Liang Lou, Zhong-Xiu Wu, Jin-Qin Jiang, Xing-Li Fan, Li-Fang Wang, Xiao-Xiang Liu, Peng Du, Jie Yan and Ai-Hua Sun

Abstracto

Trasfondo: En el diagnóstico clínico se necesitan urgentemente diagnósticos rápidos de la tuberculosis pulmonar con frotis negativa y de la tuberculosis extrapulmonar. Nuestra investigación tiene como objetivo investigar la utilidad del ensayo de liberación de interferón-γ para el diagnóstico de TB pulmonar y extrapulmonar con frotis negativa.
Métodos: Se realizaron pruebas de anticuerpos contra la tuberculosis y TB-IGRA en 389 pacientes con TB pulmonar (incluidos 120 pacientes con tuberculosis pulmonar con frotis positiva y 269 con TB pulmonar con frotis negativa), 113 pacientes con TB extrapulmonar, 81 con otras enfermedades pulmonares y 100 controles sanos . Se recogieron, procesaron e interpretaron muestras de sangre para la prueba de TB-Ab y la TB-IGRA de acuerdo con el protocolo del fabricante.
Resultados: La relación de detección de pacientes con TB pulmonar con frotis positiva y TB pulmonar con frotis negativa fue de 90.8% (109 de 120) y 89.6% (241 de 269), respectivamente. No hubo diferencias estadísticamente significativas de su desempeño entre estos dos conjuntos de muestras (P> 0.05). La proporción de detección de pacientes con tuberculosis positiva y extrapulmonar fue 90.0% (350 de 389) y 87.6% (99 de 113) respectivamente, lo que no fue significativamente diferente (P> 0.05).
Conclusiones: En este trabajo, la tasa de detección total utilizando TB-IGRA fue del 89.4%, por lo tanto, la TB-IGRA tiene valores diagnósticos en la TB pulmonar con frotis negativa y el diagnóstico de TB extrapulmonar.

Translated from English version into French by Laura C Vicente Rodriguez
